# Optimizing existing health systems: an argument for integrating functioning information

**DOI:** 10.1186/1472-6963-14-S2-P56

**Published:** 2014-07-07

**Authors:** Maren Hopfe, Birgit Prodinger, Jerome Bickenbach, Alarcos Cieza, Gerold Stucki

**Affiliations:** 1 Swiss Paraplegic Research, Nottwil, Switzerland; 2 Department of Health Sciences & Health Policy, University of Lucerne, Lucerne, Switzerland; 3 Faculty of Social and Human Sciences, School of Psychology, University of Southampton, Southampton, UK

## Background

Health systems are being challenged, in meeting the needs of growing numbers of people living with chronic health conditions, where cure of the disease is not the primary outcome of care but rather maintaining or improving functioning over a person’s life span. Functioning is an umbrella term; it encompasses body structures and body functions, as well as people’s capacity and actual performance to conduct activities of daily living and to participate in society. It is a multi-dimensional and interactive concept, which describes not merely the consequences of health, but constitutes a fundamental component for understanding health and how it plays out in everyday life.

Health information constitutes the foundation for any evidence-based decision making related to finances, service delivery, policy and governance in health systems. Though, there is agreement that disease-specific and functioning information are conceptually complimentary, health systems could be further optimized by accounting for health and how it plays out in everyday life on an operational level. If not, current health systems will continue to fail in predicting e.g. length of stay, discharge destination, and service costs of people with chronic health conditions. Ultimately, they will be limited in providing optimal care for this growing sub-population. The aim of this paper is to describe how functioning information can be integrated systematically in health systems.

## Materials and methods

This conceptual paper builds upon the six building blocks of the World Health Organization’s (WHO) systems’ framework to examine systematically the integration of functioning information in health systems. Health information is one of the building blocks and at the same time is an essential element for the other building blocks (leadership and governance, financing, medical products, vaccines and technologies, service delivery, and health workforce). Examples outlining the need, added value, and challenges of integrating functioning information in each block will be shown.

## Results and conclusions

The examples highlight that domains of functioning are recognized and considered also on the operational level of health systems. However, one of the big challenges within and across all the building blocks is the lack of conceptual clarity and consistency on what defines functioning. Although standards exist, it remains that they need to be implemented to ensure that information on functioning, in addition to disease-specific information, is available for evidence-based decision making. It can be concluded that dialogue amongst stakeholders within and beyond health systems is central to agree upon the systematic implementation of standards.

